# Understanding Hemodialysis-Associated Pericarditis: Causes, Symptoms, and Management Strategies

**DOI:** 10.3390/jcm14175944

**Published:** 2025-08-22

**Authors:** Ileana Peride, Ana-Maria Nechita, Bianca Dumitrache, Mirela Tiglis, Tiberiu Paul Neagu, Ionel Alexandru Checherita, Andrei Niculae

**Affiliations:** 1 Clinical Department No. 3, Carol Davila University of Medicine and Pharmacy, 020021 Bucharest, Romania; ileana_peride@yahoo.com (I.P.); niculaeandrei@yahoo.com (A.N.); 2 Department of Nephrology and Dialysis, St. John Emergency Clinical Hospital, 042122 Bucharest, Romania; 3 Marie Skłodowska Curie Children Emergency Clinical Hospital, 077120 Bucharest, Romania; 4 Department of Anesthesia and Intensive Care, Emergency Clinical Hospital of Bucharest, 014461 Bucharest, Romania; 5 Clinical Department No. 11, Carol Davila University of Medicine and Pharmacy, 020021 Bucharest, Romania; 6 Department of Nephrology, Ilfov County Emergency Clinical Hospital, 022104 Bucharest, Romania; al.checherita@gmail.com

**Keywords:** hemodialysis, pericarditis, clinical outcome, treatment

## Abstract

Hemodialysis-associated pericarditis is a significant but insufficiently acknowledged complication in patients with end-stage renal disease (ESRD). It can manifest as either uremic pericarditis, typically occurring before or shortly after the initiation of dialysis, or dialysis-associated pericarditis, which results from prolonged dialysis treatment. The condition is associated with substantial morbidity and potential mortality due to risks, such as cardiac tamponade and constrictive pericarditis. Pericardial involvement in ESRD most frequently presents as acute uremic or dialysis-associated pericarditis, whereas chronic constrictive pericarditis represents a less common manifestation. The aim of the article is to review the current understanding of the epidemiology, pathophysiology, clinical presentation, diagnostic criteria and therapy strategies of this pathology based on a case of hemodialysis-associated pericarditis in a patient diagnosed with sudden shortness of breath during a hemodialysis session. When assessing pericarditis in this group of population, it is recommended to distinguish between uremic and dialysis-associated forms, to recognize clinical warning signs, and to customize the treatment. Probably the therapy should include anti-inflammatory drugs, colchicine, intensified dialysis, and in severe cases, even pericardiocentesis or surgical intervention. Rising awareness and timely intervention are critical to improve outcomes in this vulnerable population.

## 1. Introduction

End-stage renal disease (ESRD), the final stage of chronic kidney disease associating a glomerular filtration rate less than 15 mL/min/1.73 m^2^, is associated with a range of cardiovascular complications, including both myocardial and pericardial disorders [1]. Among these, pericardial diseases, such as pericarditis, pericardial effusion, and cardiac tamponade, can be encountered in patients with kidney failure. Despite their prevalence, there is limited evidence to guide the optimal management of these conditions, particularly in the dialysis population [2].

In a healthy pericardium, in order to preserve a normal lubrification, 15–50 mL fluid is present between the visceral and parietal layers [1]. In special conditions, such as infection, malignancy, autoimmune pathologies, uremia, pericardial inflammation and increased microvascular permeability can occur, representing the main triggers of pericardial effusion onset [1,3]. Pericardial effusion is present in 6.5% of the general population and usually can be asymptomatic. The major concern is the presence of this pathology in high-risk patients that often associate a complex form which can progress to cardiac tamponade. Therefore, a rapid diagnosis and adequate therapy management are required in order to prevent the development of this life-threatening condition associated with major hemodynamic instability [3].

Before advancement of renal dialysis and transplantation, the diagnosis of pericarditis in patients with ESRD was often a predictor of imminent mortality, typically occurring within two weeks of onset. The introduction of renal replacement therapies has significantly reduced the incidence of pericarditis and its associated complications. However, pericardial involvement continues to represent a notable source of morbidity and mortality among patients with ESRD [2].

In patients undergoing hemodialysis (HD), the presence of pericarditis requires thorough evaluation. Uremic pericarditis refers to cases that occur before or within the first eight weeks of initiating dialysis [2], whereas dialysis-associated pericarditis, including constrictive forms, is diagnosed when clinical features emerge after patients have been stabilized on dialysis for at least eight weeks [4].

In HD patients, it seems that pericarditis is not usually caused by traditional risk factors as in general population, but due to an inadequate dialysis therapy which can lead to important toxins accumulation. Consequently, this increase in uremic toxic metabolites determines an important release of several pro-inflammatory markers responsible for pericardium inflammation and, later on, fibrous depositions [1].

Considering the complexity of this condition, especially in HD patients, we conducted a review of the current literature database regarding the current knowledge of the epidemiology, pathophysiology, clinical presentation, diagnostic criteria and therapy strategies of hemodialysis-associated pericarditis. In order to emphasize the importance of this topic, and the optimal therapy management, we considered appropriate to describe the case of a HD patient presenting sudden shortness of breath during a hemodialysis session.

## 2. Review of Literature

### 2.1. Epidemiology

There are few epidemiological data regarding pericarditis in ESRD patients, but with the improvement of dialysis technique and a better assessment of ESRD population, it seems the incidence decreased; recent data reported an incidence of uremic pericarditis between 1.4% and 29% per year, especially in male patients, and for dialysis-associated pericarditis between 0.8% and 6% [5,6].

The incidence of uremic pericarditis has declined to approximately 5–20% among patients undergoing chronic hemodialysis, a trend that may be attributed to advancements in the efficacy of hemodialysis treatment [5,6,7]. However, the incidence of hemodialysis-related pericarditis may rise due to the increased number of patients on chronic dialysis.

A French study on 46 patients aged 46 ± 25.7 years with a male/women report = 2.06 observed 13 cases of pericarditis (28.2%) after a recoil of 14.7 months (1–27 months), which confirms the progression of inflammation and fibrosis in hemodialysis patients [8].

Pericarditis occurs due to the inadequacy of dialysis in stable ESRD patients or relatively inefficient dialysis in patients with higher catabolic activity or with different vascular access dysfunctions [1,4,9].

### 2.2. Etiology and Classification

Pericarditis in chronic dialysis patients can result from multiple mechanisms, typically related to the underlying effects of kidney failure and dialysis-related factors. The main contributing mechanisms include disturbances in fluid and electrolyte balance resulting in the accumulation of toxic metabolites, such as nitrogenous wastes, volume overload, inadequate dialysis dose (underdialysis) and inflammatory response to bio-incompatible dialysis membranes [10].

There are studies concluding that an increase in nitrogen waste products has a proinflammatory effect leading to pericarditis; others have reported that the changes in acid-base homeostasis, hypercalcemia, and hyperuricemia are all implicated in the development of uremic pericarditis [1,5,6].

Infectious pericarditis, caused by immunosuppression and indwelling catheters, increases the risk of infection. The pathogens involved could have bacterial (especially *Staphylococcus* spp.), viral, or fungal origin. Infection spreads to the pericardium via hematogenous pathway or contiguous infection. The risk is higher in patients on peritoneal dialysis or with catheter-related bloodstream infections [11]. Autoimmune and inflammatory disorders are also responsible for pericarditis in chronic dialysis population. Some patients may have systemic autoimmune diseases (e.g., systemic lupus erythematosus), which can independently cause pericarditis. Uremia may also alter immune function, contributing to chronic low-grade inflammation. Post-pericardiotomy or post-myocardial injury syndrome (Dressler’s-like) is rare in dialysis patients, but possible after cardiac surgery or infarction and involves autoimmune reaction against pericardial antigens [1,12,13,14,15,16,17,18,19,20].

Depending on duration and morphology, pericarditis can be classified as acute and chronic, including different types, such as fibrinous, purulent, hemorrhagic etc. (Table 1) [1,12,13,14,15,16,17,18,19,20].

### 2.3. Pathogenesis

Pericarditis in chronic dialysis patients can be classified as either uremic pericarditis or dialysis-associated pericarditis, depending on the timing relative to dialysis initiation. Uremic pericarditis typically occurs before or within the first 8 weeks of dialysis, whereas dialysis-associated pericarditis develops after 8 weeks or more of maintenance dialysis [1,3].

The pathophysiology of pericarditis in these patients is multifactorial. In uremic pericarditis, the accumulation of toxic metabolites due to inadequate renal clearance is a central mechanism. Uremic toxins (although not fully identified) are believed to trigger a proinflammatory response within the pericardial layers, leading to fibrinous or serofibrinous inflammation [21]. The accumulated uremic toxins contribute significantly to this inflammatory environment. They can activate immune cells (like macrophages) and trigger the release of pro-inflammatory cytokines, such as interleukin-1 (IL-1), interleukin-6 (IL-6) and tumor necrosis factor-alpha (TNF-α). These cytokines then initiate and propagate inflammation in the pericardium. This inflammatory process often involves infiltration by mononuclear cells and may extend into the subepicardial myocardium [21,22]. Oxidative stress, another hallmark of uremia, contributes to endothelial dysfunction and cell injury, further exacerbating the inflammatory response [21,23].

In ESRD, the kidneys fail to adequately excrete metabolic waste products and toxins. These “uremic toxins” (including urea, creatinine, and other compounds) build up in the bloodstream [21,23]. These toxins are thought to directly irritate and damage the delicate tissues of the pericardium (the sac surrounding the heart). This leads to an inflammatory response [1,3].

Studies suggest that elevated levels of BUN (blood urea nitrogen), typically above 60 mg/dL, are correlated with uremic pericarditis, although the severity of pericarditis does not always directly correlate with BUN or creatinine levels [21,23].

Electrolyte imbalances, such as hypercalcemia and hyperuricemia, along with acid-base disturbances, have also been identified as contributing factors. These abnormalities may exacerbate the inflammatory environment within the pericardial space [1,6,24].

In dialysis-associated pericarditis, the condition is frequently linked to inadequate dialysis therapy, either due to reduced dialysis efficiency or missed sessions, often secondary to different vascular access pathologies [1,25,26]. Patients with high catabolic activity secondary to various comorbid conditions may experience relatively insufficient clearance of inflammatory mediators, even with an adequate dialysis [26,27].

In both uremic and dialysis-associated pericarditis, an inefficient dialysis is a major contributing factor. It can be caused by insufficient dialysis sessions or not long enough to effectively clear toxins, poor adherence to dialysis schedules (e.g., patients missing treatments), dysfunctions of vascular access that lead to inefficient toxin removal and increased catabolic state, patients with severe underlying illnesses or infections that may generate a higher amount of toxins incompletely removed by their regular HD regimen [26,27,28].

An optimal dialysis schedule along with a compliant patient represent the cornerstone of treatment because there is probably a direct link between the toxin accumulation and inflammatory process [26,27,28].

Additionally, volume overload can contribute to pericardial effusion in dialysis patients, which may not always have an inflammatory origin but can lead to pericardial irritation and subsequent inflammation [26,27,28].

Patients with uremia have a higher risk of bleeding due to platelet dysfunction and alterations in the coagulation cascade. This can contribute to hemorrhagic pericardial effusions in some cases, especially in dialysis-associated pericarditis where anticoagulation (e.g., heparin) is used during hemodialysis [1,26,29,30].

Though uncommon, infectious pericarditis (bacterial, viral, or fungal) should also be considered, particularly in immunocompromised patients or those with indwelling catheters [1,26,29,30].

The pathology of dialysis-associated pericarditis differs from that of uremic pericarditis, notably in the typical composition of the pericardial fluid, which is often serosanguinous [1,26,29,30]. Prolonged adhesive pericarditis with intermittent hemorrhage may predispose patients to subacute constrictive pericarditis, a complication that can emerge relatively soon after an episode of acute pericarditis. Over time, continued inflammation and hemorrhage may result in progressive fibrosis, ultimately leading to chronic constrictive pericarditis. Notably, the incidence of chronic constrictive pericarditis has increased since the introduction of dialysis, with 3.7% to 12% of cases progressing to constriction [26], likely reflecting improved survival rates among patients with uremic pericarditis due to the availability of renal replacement therapy [1,21,31].

Cardiac tamponade, a life-threatening complication of pericarditis, is notably more prevalent in dialysis patients. The probability of developing cardiac tamponade in a patient with pericarditis who is not on dialysis is approximately 3.1%. In contrast, this rate increases to approximately 10–20% in patients undergoing dialysis [32].

**Figure 1:**
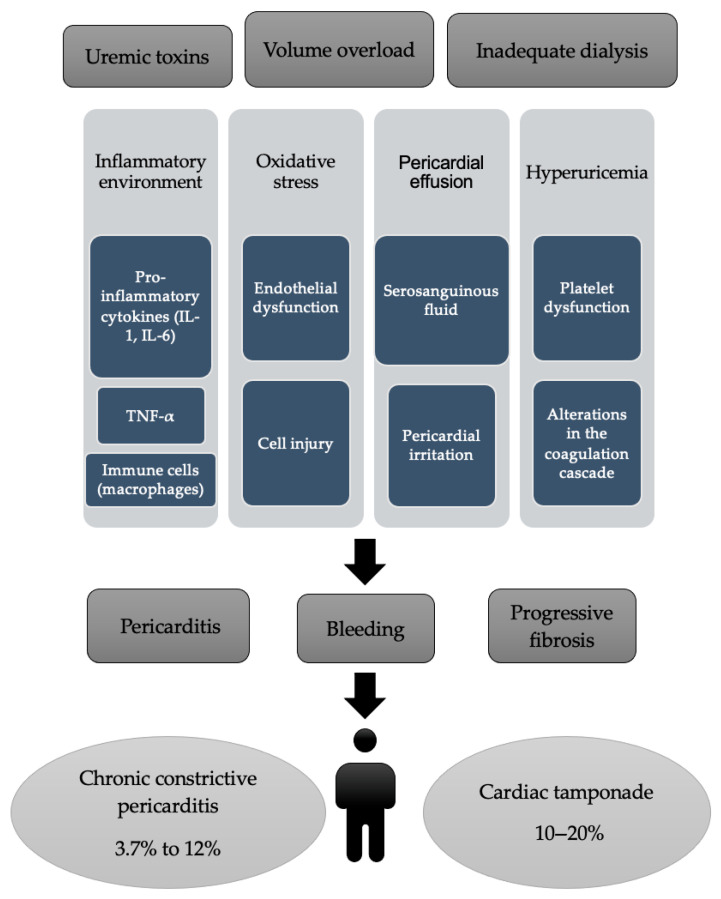
The main causes of pericardial effusion.

Therefore, while pericarditis remains a significant concern in ESRD patients, the risk of progression to cardiac tamponade is substantially higher in those on dialysis, underscoring the importance of vigilant monitoring and timely intervention in this population [33]. Figure 1 summarizes the most important causes of pericardial effusion [1,3,6,21,22,23,24,25,26,27,28,29,30,31,32,33].

### 2.4. Diagnosis

ESRD-associated pericarditis can be divided into three groups: uremic, dialysis-associated, and mixed.

Symptoms include chest pain that is common; dyspnea, fever, chills, malaise, weight loss and pericardial rub are frequent, as well. Cardiac tamponade is reported in 5% of cases. Gallop rhythm, jugular venous distention, hepatomegaly and hypotension are also common [1,5,7,26,30,34,35,36,37,38].

Signs of cardiac tamponade include orthopnea, hypotension, jugular vein distension (JVD), pulsus paradoxus, muffled heart sounds [4,26,30,31,35].

Chronic constrictive pericarditis presents with systemic congestion (JVD, hepatomegaly, ascites, peripheral edema) and a pericardial knock [4,26,30,31,35].

Laboratory and imaging findings disclose leukocytosis and elevated erythrocyte sedimentation rate (ESR) are typical [1,5,7,26,30,34,35,36,37,38].

Cardiomegaly and abnormal cardiac silhouette on chest X-ray are common [4,7,26,30,31,33,34,35,36,37,38].

On electrocardiogram (ECG), classic pericarditis patterns (ST elevation, PR depression) are uncommon; nonspecific changes are more typical due to LVH (left ventricular hypertrophy) or electrolyte disturbances. Arrhythmias, especially atrial fibrillation, may be noticed [7,26,30,31,33,34,35,36,37,38]. Electrocardiographic findings of acute pericarditis consist in 4 stages [38]:

1. Stage I—diffuse modifications, such as ST-segment augmentation and PR-segment elevation in aVR-lead and V1 (differential diagnosis with myocardial infarction);
2. Stage II—normalization of ST- and PR-segments, in the first week;
3. Stage III—T-wave inversion;
4. Stage IV—T-wave normalization.

Echocardiography is the key tool to detect and assess pericardial effusion, which may be due to volume overload, not just pericarditis. Ultrasound helps evaluate effusion size, location and signs of tamponade (e.g., right ventricle diastolic collapse) [3,6,26,30,31,33,34,35,36,37,38]. Based on the echocardiography findings, pericardial effusion volume can be calculated with squared formula for effusion volume (an estimative method) [39]:

Volume (mL) = (maximum echo − free space in mm)^2^ × 0.7

### 2.5. Complications

The major life-threatening complication of hemodialysis-related pericarditis is the development of cardiac tamponade. If a rapid or excessive fluid accumulates in the pericardial sac, it has the potential to compress the heart, making it unable to pump blood to the rest of the body. A typical presentation of cardiac tamponade includes hypotension, tachycardia, increased jugular venous distension, pulsus paradoxus, and muffled heart sounds. An electrocardiogram may show alternating QRS amplitudes in any or all leads—which is a discrepancy in voltage caused by the heart floating within the pericardium due to the effusion. The treatment for this complication is pericardiocentesis or surgical drainage [40].

Constrictive pericarditis is defined as chronic thickening and scarring of the pericardium, which restricts diastolic filling of the heart and can be also caused by recurrent or prolonged inflammation (often post-dialysis pericarditis). Symptoms include fatigue, peripheral edema, ascites, hepatomegaly, and elevated jugular venous pressure. It often requires pericardiectomy [40].

Another complication is hemorrhagic pericardial effusion, which describes blood in the pericardial sac, sometimes due to anticoagulation or uremia-related platelet dysfunction. Risk factors include the use of heparin during dialysis or systemic anticoagulation. Diagnosis should not be missed due to the increased risk of tamponade and poor outcomes [40].

Infectious pericarditis occurs secondary to contamination of the pericardial sac or fluid by bacterial (e.g., *Staphylococcus aureus*), viral or fungal pathogens, typically introduced during invasive medical procedures or as a result of vascular access-related infections, particularly in immunosuppressed patients [40].

Dialysis interruptions have a serious impact on the patient’s health because severe pericarditis may result in the temporary cessation of dialysis due to hemodynamic instability, further worsening uremia and metabolic imbalance [40].

### 2.6. Treatment

Various therapeutic approaches have been employed in the management of uremic and dialysis-associated pericarditis, as well as related pericardial effusions. These include intensive hemodialysis, peritoneal dialysis, oral administration of nonsteroidal anti-inflammatory drugs (NSAIDs), oral glucocorticoid therapy, pericardiocentesis, intrapericardial instillation of a long-acting, nonabsorbable glucocorticoid, pericardiostomy, creation of a pericardial window, and, in selected cases, pericardiectomy [41]. Nevertheless, all medical therapies for pericardial diseases are off-label, since no drug has been registered until now for a specific pericardial indication [41].

Intensive hemodialysis is typically defined as daily dialysis sessions over a 10- to 14-day period. Its reported efficacy in the treatment of uremic pericarditis ranges from 76% to 100%, with most studies indicating a success rate of approximately 85%. Recurrence of pericarditis following this treatment occurs in up to 15% of cases. Despite this, the recurrence rate is considered acceptable when compared to the high mortality rate (estimated between 88% and 100%) associated with untreated uremic pericarditis. In contrast, the role of peritoneal dialysis in managing uremic pericarditis remains unclear, as it has not been extensively studied for this indication [4,7,26,31,41,42,43].

Aspirin and nonsteroidal anti-inflammatory drugs (NSAIDs) represent the cornerstone of treatment for acute pericarditis. Drug selection should be individualized based on the patient’s medical history, including any contraindications, prior treatment responses, and adverse effects. Consideration should also be given to comorbid conditions; for example, aspirin may be preferred over other NSAIDs in patients who require antiplatelet therapy. Additionally, physician experience and clinical judgment play a key role in guiding appropriate therapy [41,44,45,46,47].

Colchicine is recommended at low, weight-adjusted doses to enhance the effectiveness of medical therapy and reduce the risk of recurrence [41,48,49,50,51,52,53,54]. While tapering of colchicine is not strictly necessary, it may be considered to minimize the persistence or return of symptoms. Corticosteroids should be reserved as a second-line option for patients with contraindications to, or inadequate response from, aspirin or NSAIDs, due to their association with increased risk of chronic disease progression and potential for dependency. When corticosteroids are indicated, they should be administered in conjunction with colchicine. Low to moderate doses, such as prednisone at 0.2–0.5 mg/kg/day or its equivalent, are preferred over high-dose regimens (e.g., prednisone 1.0 mg/kg/day) [41,50]. The initial dose should be continued until clinical symptoms resolve and C-reactive protein levels normalize, after which a gradual taper should be initiated [41,48,49,50,51,52,53,54].

Management of pericardial effusion should be guided primarily by the underlying etiology. In approximately 60% of cases, the effusion is secondary to an identifiable systemic or cardiac condition, and treatment should focus on addressing the primary disease. When pericardial effusion is associated with pericarditis, the therapeutic approach should align with established pericarditis management protocols. In instances where the effusion becomes symptomatic in the absence of overt inflammation, or when empiric anti-inflammatory therapy proves ineffective, pericardial drainage should be considered [41,55,56,57].

Pericardiocentesis, accompanied by extended drainage (up to 30 mL/day), may facilitate the apposition of pericardial layers and reduce the likelihood of fluid re-accumulation. However, this strategy is primarily supported by evidence from case reports, retrospective analyses, and expert consensus [41,55,56,57].

Currently, no medical therapies have been definitively proven to reduce isolated pericardial effusions. In non-inflammatory effusions, agents such as NSAIDs, colchicine, and corticosteroids are generally ineffective [41,52,57,58]. In such cases, pericardiocentesis may be necessary for symptom relief and resolution of large effusions. Nonetheless, recurrence is not uncommon, and in cases of reaccumulating, loculated, or diagnostically ambiguous effusions, more definitive interventions such as pericardial window creation or pericardiectomy should be considered [41,55].

The utilization of both pericardial window and pericardiectomy has demonstrated excellent outcomes in patients with end-stage renal disease (ESRD) presenting with refractory, symptomatic, and/or hemodynamically significant pericardial effusion [52].

Rutsky and Rostand reported a 100% success rate in a small cohort of three patients with dialysis-associated pericarditis and pericardial effusion who underwent pericardial window procedures [42].

**Figure 2:**
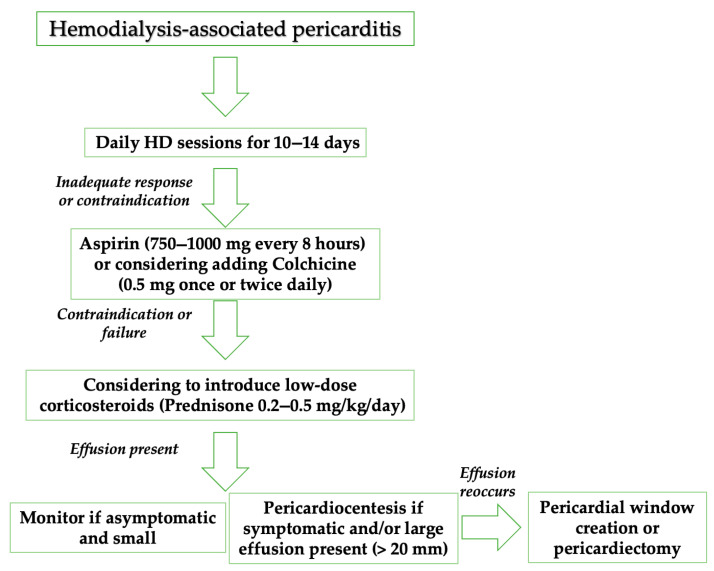
Therapy management of uremic and dialysis-associated pericarditis. HD: hemodialysis.

In Figure 2, we summarize the therapy options for pericarditis in predialysis and HD patients [41,42,43,44,45,46,47,48,49,50,51,52,53,54,55,56,57,58].

## 3. Case Presentation

A 70-year-old man on maintenance dialysis three times a week, presented sudden shortness of breath, headache and fatigue during a hemodialysis session. For 11 years, he performed chronic dialysis with a left brachiocephalic arteriovenous fistula, but for more than three months, deliberately, the patient completed shorter dialysis sessions (up to 3-h). His medical records showed a complex association of comorbidities, such as hypertensive cardiopathy, ischemic cardiomyopathy, pulmonary hypertension, mitral regurgitation, degenerative aortic stenosis, chronic obstructive pulmonary disease, restrictive lung disease, diabetes type 2 with insulin therapy, secondary hyperparathyroidism, secondary anemia, dyslipidemia and benign prostatic hyperplasia.

At admission, the chest x-ray showed pleural fluid accumulation on the left side in medium–large quantity and minimal pleural fluid accumulation on the right side. The native CT scan revealed pericardial fluid accumulation, up to 33 mm with cardiomegaly, left basal pulmonary condensation, 23 mm left pleurisy and minimal right pleurisy.

Post-dialysis laboratory tests showed increased nitrogenous waste products (serum creatinine = 6.99 mg/dL, urea = 127.1 mg/dL), metabolic acidosis, hyperkalemia, hyperglycemia, high levels of aspartate aminotransferase, inflammatory syndrome (C-reactive protein = 223.6 mg/L, fibrinogen = 759 mg/dL), leukocytosis (13,000 µL), mild anemia (Hb = 9.4 g/dL), international normalized ratio (INR) = 1.48 without anticoagulant treatment. Admission ECG showed sinus rhythm and incomplete right bundle branch block. ST-segment elevations were observed in the limb leads (DII, DIII, aVF) and precordial leads (V2–V3), accompanied by negative T wave in V1–V3.

**Figure 3:**
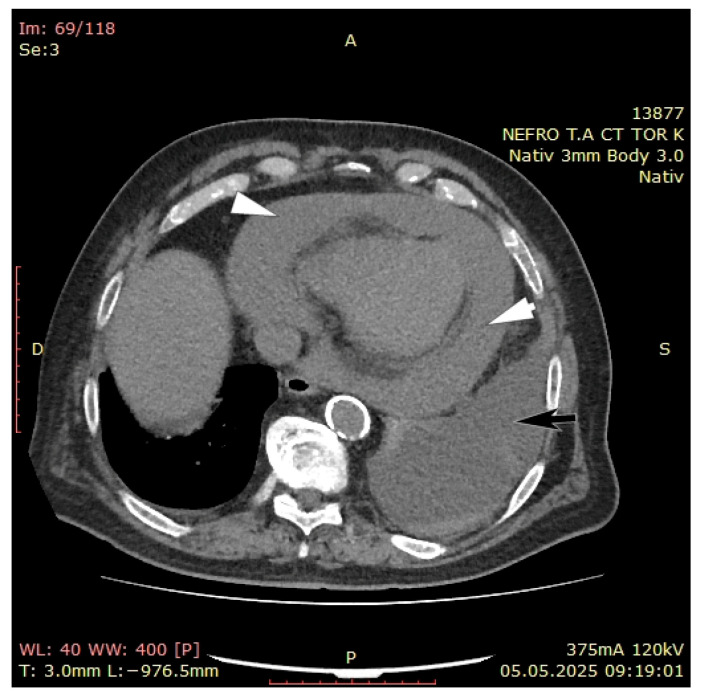
CT with contrast agent revealing important pericardial (white arrows) and pleural effusion (black arrow).

On the fifth day, even after adequately performed dialysis sessions, his condition continued to deteriorate, presenting hypotension and chest pain, along with increased oxygen therapy requirement. A computed tomography (CT) with contrast agent was performed that showed important pericardial effusion (43 mm) and pleural effusion on the left side (40 mm) (Figure 3).

**Figure 4:**
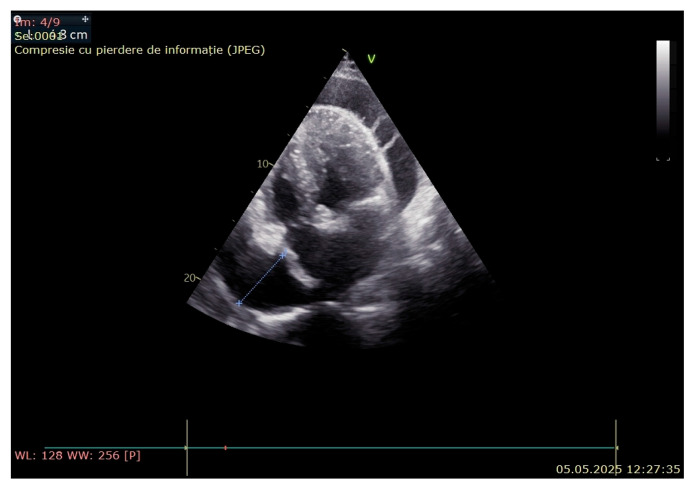
Echocardiography revealed right atrium collapse and massive pericardial fluid.

A cardiological assessment was performed due to his large pericardial effusion and the risk of cardiac tamponade. Echocardiography (General Electric Vivid^TM^ E95 edition, Chicago, IL, USA) revealed right atrium collapse, massive pericardial fluid, calcifications of the anterior and posterior mitral valve and hyperechogenic image on the slope of the anterior mitral valve leaflet of 2.5 mm—possible vegetation on the atrial side of the mitral valve (Figure 4). Transesophageal echocardiography could not be performed, because this examination was not available in our hospital.

Considering the high risk of cardiac tamponade, pericardiocentesis was performed through a left subxiphoid approach with angiographic guidance, using the modified Seldinger technique under aseptic conditions, and without any complications. 1300 mL of hemorrhagic fluid was evacuated and the sample was sent to the laboratory (amount in accordance with the squared formula for effusion volume (43)^2^ × 0.7 = 1849 × 0.7 = 1294 mL). Analysis of the pericardial effusion identified low levels of albumin and serum proteins and high lactate dehydrogenase levels with normal levels of amylase without positive aerobic, anaerobic and fungal cultures. Cytological examination revealed scattered mesothelial cells on an inflammatory background, characterized by the presence of polymorphonuclear cells, histiocytes, and a hemorrhagic component.

On the same day, during the HD session, the patient presented gastrointestinal bleeding, manifested by melena with important drop of hemoglobin levels (initially, 9.4 g/dL, then 6.9 g/dL); he received blood transfusion, proton pump inhibitors therapy and a nasogastric tube was inserted for 24-h. An upper digestive endoscopy with anesthesia was performed that showed ulcerative lesions on the duodenal bulb and pyloric antrum, and venous bleeding. 4 hemoclips were applied to achieve hemostasis.

Due to elevated transaminases levels, colchicine therapy was delayed, and the treatment was started on day 20 (1 mg per day). In our patient, NSAIDs and prednisone could not be considered due to the presence of ulcers and the possibility of an infectious state. In addition, daily dialysis sessions were not applied as the patient was performing chronic dialysis with a left brachiocephalic arteriovenous fistula and he presented a favorable clinical evolution (oxygen demand gradually decreased, and dyspnea improved).

**Figure 5:**
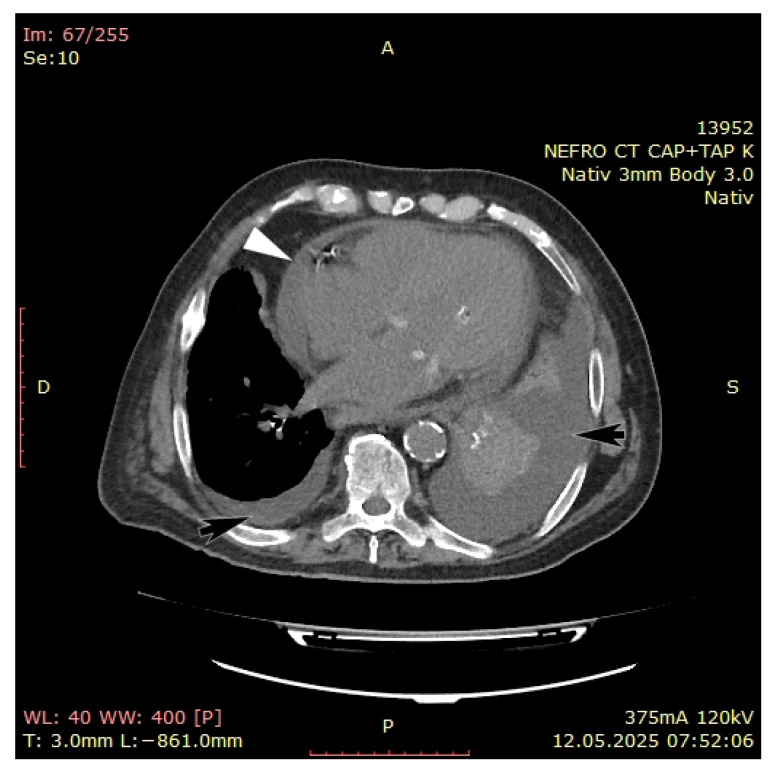
Follow-up CT scan with contrast agent highlighted an accumulation of fluid of 18 mm adjacent to the right atrium (white arrow); pleurisy of 14 mm on the right side and 3 mm on the left side (black arrows).

The follow-up tomography revealed an accumulation of fluid of 18 mm adjacent to the right atrium, small mediastinal adenopathy of about 15/13 mm, complete atelectasis of the lower left lung lobe with intrabronchial fluid accumulations and bilateral pleurisy of up to 14 mm on the right side and 3 mm on the left side, respectively. No expansive nodular lung lesions with tumor substrate were visualized (Figure 5).

During the following days the patient presented another episode of melena stools simultaneous with a slight decrease in the Hb level (Hb = 8.1 g/dL), and a second upper digestive endoscopy was performed that revealed gastroduodenitis and correctly positioned prepyloric clips, without active bleeding or digested blood in the stomach. Colonoscopy showed external hemorrhoids and several non-neoplastic polyps.

Considering the gastrointestinal pathologies, HD sessions were limited to 3 h without anticoagulation and blood transfusions were performed.

Follow-up echocardiography showed decreased pericardial fluid compared with the initial assessment (8 mm adjacent to the right atrium) and tight aortic stenosis with calcifications and possible attached valvular formation.

Although the echocardiography findings suggested the possibility of vegetation and infective endocarditis was initially considered, the diagnosis was ultimately ruled out due to negative blood cultures and the absence of other supporting diagnostic criteria. Cancerous pericarditis was considered less likely because histopathological examination of the pericardial fluid was negative for malignancy, the blood tests were negative for tumor markers and no apparent tumor lesions were noted on CT assessment. In addition, viral and myocardial infectious pericarditis were considered less likely, as viral tests were negative. Consequently, dialysis-associated pericarditis was considered the most probable diagnosis.

On 23rd day, the patient presented a high ventricular rate and the ECG revealed atrial fibrillation. The cardiac assessment recommended blood transfusion to correct anemia, and initiation of anticoagulant treatment when the clinical-biological condition would allow it.

When gastrointestinal bleeding was excluded, minimal dose of anticoagulant was initiated. Once the patient was able to resume dialysis on schedule (4-h sessions) and the hemoglobin levels were stabilized, he was discharged with the following recommendations: 30 days of colchicine administration and echocardiography assessment, after 1 month.

**Figure 6:**
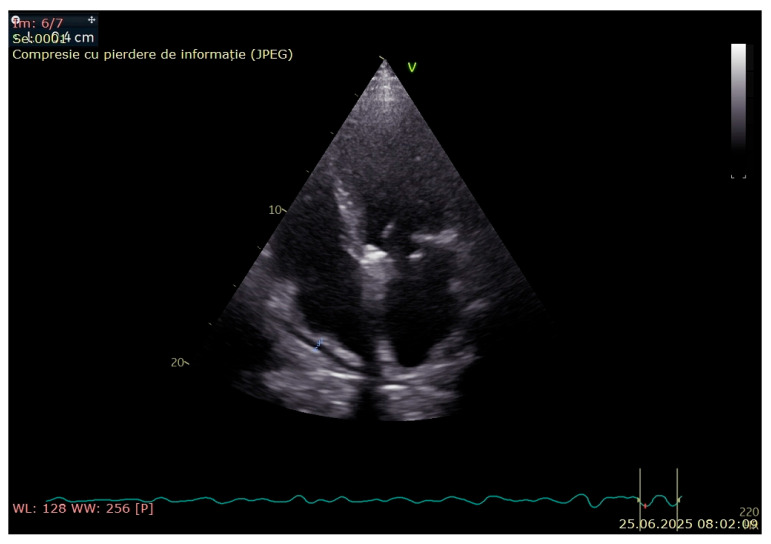
The 1-month follow-up echocardiography.

After 1 month, the follow-up echocardiography showed decreased pericardial fluid compared with the last assessment (4 mm adjacent to the right atrium), aortic valve with calcifications, moderate-severe aortic stenosis (peak aortic jet maximum > 4.5 m/s, gradient maximum > 65 mmHg), moderate aortic regurgitation, anterior mitral valve/posterior mitral valve (VMA/VMP) with calcifications and left atrium > 60 mm (Figure 6). The recommendations were to continue with colchicine only for another 30 days (with a decreased dose of 0.5 mg per day), and to be evaluated by a cardiac surgeon, due to presence of aortic valve calcifications associated with moderate-severe aortic stenosis. Furthermore, taking into account the link between valvular aortic stenosis and acquired von Willebrand factor deficiency may have also played a role in the development of hemorrhagic pericarditis [59] valve replacement probably will offer the best chance for long-term resolution in our patient.

## 4. Discussion

Due to the limited available data, we aimed to perform a review of ESRD-associated pericarditis that presents high risk of morbidity and mortality if it is not diagnosed on time and the treatment is not immediately initiated. In order to improve long-term prognosis and patients’ quality of life, based on our expertise and current knowledge, a multidisciplinary approach is required in cases associating voluminous pericarditis secondary to underdialysis.

According to several studies, if the onset of uremic pericarditis is caused by several metabolic alterations, such as hyperuricemia, hyperparathyroidism, hypoproteinemia, hyperkalemia and toxic metabolites accumulation, HD-associated pericarditis development is mainly linked to inadequate dialysis [5,60]. Uremic toxins accumulations represent an important trigger not only for a major pro-inflammatory response, but for a higher risk of bleeding secondary to several coagulation disorders (e.g., platelet dysfunction, fibrinolytic system activation, altered coagulation cascade), probably explaining the development of ulcerative lesions in our patient. Nevertheless, studies failed to show a clear correlation between the degree of azotemia and pericarditis onset in ESRD patients [60]. A recent study showed that NLRP3 (nucleotide-binding oligomerization domain-like receptor protein 3) inflammasome activation, responsible for interleukin 1 release, may be incriminated in the development of pericarditis. The results highlighted also that several therapies (e.g., NLRP3 inflammasome inhibitor, anakinra, interleukin-1 trap, and colchicine), can successfully inhibit the activation of this pathophysiological mechanism [61]. These findings were validated by our case, considering the decrease in pericardial fluid to 4 mm after 1 month of colchicine therapy.

A study including 132 HD patients observed that echocardiography examination performed in the first 8 weeks since dialysis initiation can be beneficial and cost-effective in evaluating the efficiency of HD and to determine on time the presence of pericarditis and cardiac tamponade [62]. Considering the complexity of our case, these conclusions perhaps should be extended also in long-term chronic HD population, and an annually echocardiography examination would be required.

Nevertheless, in 2023, a case of heparin-induced hemorrhagic pericarditis was reported in a chronic HD male patient, highlighting the lack of data and conflicting evidence regarding the optimal anticoagulation dosing in this group of patients [63]. One study including 453 patients noticed a clear risk of hemorrhagic pericarditis and cardiac tamponade with anticoagulation [63,64]. In contrast, two other studies performed on 274 patients, and 822 patients, respectively, did not find any association [63,64,65]. Furthermore, the recent KDIGO (Kidney Disease: Improving Global Outcomes) guidelines failed to establish the adequate dosing in HD patients in need of permanent anticoagulation therapy [66]. Our patient presented an impressive quantity of hemorrhagic pericardial fluid, but, in contrast with the other case, he was not compliant to the HD treatment and was not receiving any anticoagulation therapy, except the anticoagulation performed during his dialysis sessions. It is clear that the exact pathophysiological mechanisms involved in hemodialysis-associated pericarditis are not entirely understood, and further research is required.

## 5. Conclusions

In patients on hemodialysis, cardiac conditions should be carefully investigated. In cases with pericarditis associating high fluid accumulation, the differential diagnosis should include idiopathic, viral, increased uremia, autoimmune, and neoplasia conditions, which can be ruled out by histopathological and laboratory tests, echocardiography, and computed tomography.

According to the European Society of Cardiology guidelines, the management of pericarditis associated with renal failure includes dialysis, pericardiocentesis or surgical drainage, NSAIDs, corticosteroids, and colchicine. Clinical concern for dialysis-associated pericarditis is its life-threatening potential due to progress to constrictive pericarditis and even cardiac tamponade if not recognized and managed. When it is possible intensive hemodialysis is suggested as an attempt to eliminate the uremic toxins responsible for pericardial inflammation. In some cases, as our, intensive HD cannot be performed or this approach can be insufficient, requiring urgent pericardial drainage or pericardiectomy, followed by anti-inflammatory therapy and colchicine.

In this patient, hemodialysis-related pericarditis developed as a life-threatening complication resulting from inadequate dialysis. Delays in diagnosis and treatment could have led to additional decompensation and adverse results. The particularity of the case is represented by the amount of pericardial fluid extracted, the risks involved and the favorable outcome under colchicine therapy and adequate dialysis sessions.

In the management of chronic pericardial effusions, careful monitoring of pericardial fluid volume and the correlation between its rate of accumulation and the patients’ clinical condition are essential to ensure timely and effective intervention. Therefore, a collaborative approach among medical specialists emphasizes the importance of early diagnosis and comprehensive management to achieve successful outcomes in these particular cases.

## Figures and Tables

**Table 1:** Classification of pericarditis.

Morphology Type	Causes
Acute forms	
Serous	Viral infections, early stages of tuberculosis pericarditis etc.
Fibrinous	Uremia, systemic lupus erythematosus, rheumatic fever etc.
Purulent	Pyogenic infections
Hemorrhagic	Neoplasia, tuberculosis pericarditis, sepsis etc.
Chronic forms	
Chronic effusive	Idiopathic or secondary to any acute forms
Chronic adhesive	Stage of organization of different forms of acute pericarditis
Chronic constrictive	Tuberculosis pericarditis, secondary to cardiac surgery or radiotherapy, purulent pericarditis etc.
Caseous	Tuberculosis, fungal infections

## Data Availability

The data presented in this study are available on request from the corresponding author due to privacy reasons.
